# The Influence of Perceived Security in Childhood on Adult Self-Concept: The Mediating Role of Resilience and Self-Esteem

**DOI:** 10.3390/healthcare11172435

**Published:** 2023-08-31

**Authors:** Juan Carlos Martín Quintana, Pedro Francisco Alemán Ramos, Paula Morales Almeida

**Affiliations:** 1 Department of Education, Universidad de Las Palmas de Gran Canaria, 35001 Las Palmas, Spain; juancarlos.martin@ulpgc.es; 2 Department of Psychology, Sociology and Social Work, Universidad de Las Palmas de Gran Canaria, 35001 Las Palmas, Spain; pedro.aleman@ulpgc.es

**Keywords:** security, attachment, positive parenting, resilience, self-esteem, self-concept

## Abstract

Secure attachment, developed through consistent relationships with attachment figures in childhood, is a crucial factor in fostering healthy interpersonal relationships and a positive self-perception. Part of the positive parenting approach and the theory of affect is taken as a basis to assess how perceived security, as an indicator of secure attachment, predicts adult self-concept through the mediating effects of resilience and positive self-esteem. A quantitative, cross-sectional, and nonprobabilistic study was conducted with 383 participants. We unveiled significant positive associations between perceived security, self-concept, resilience, and positive self-esteem. Path analysis demonstrated that resilience and positive self-esteem sequentially mediate the relationship between perceived security during childhood and adult self-concept. Findings suggest that the ability to cope with adversity and personal acceptance underlie the impact of perceived security on self-concept. It is recommended to promote family intervention programs framed within positive parenting that focus on fostering secure attachment given its influence on adult life. It is also deemed essential to incorporate the promotion of resilience and self-esteem into programs aimed at youths, and adult role models can enhance their self-perception and resilience in the face of potential effects stemming from insecure parenting practices.

## 1. Introduction

The Committee of Ministers of the Council of Europe enacted Recommendation REC (2006)19 [1], urging the Member States of the European Union to implement policies that promote positive parenting. This approach focuses on parents meeting the basic needs of their children, fostering healthy, stable, and secure emotional bonds, as well as providing a well-structured family context characterized by adequate parental supervision and the establishment of clear and flexible rules [2]. However, the same Recommendation highlights other principles of positive parenting, such as stimulating and supporting children’s learning and everyday activities, promoting their capabilities, recognizing and showing interest in their world, and, above all, raising them without violence. The security and emotional attachment established through positive parenting play a crucial role in the development and wellbeing of children. This novel perspective on positive parental engagement underpins any endeavor aimed at enhancing the parental role, thereby influencing the development and education of children. Furthermore, attachment theory [3] elucidates the formation and sustenance of the attachment bond as an intrinsic motivational system for seeking emotional security, protection, and regulation during stressful situations.

For Bowlby [3] and Ainsworth [4], secure attachment is characterized by trust in the caregivers’ availability and sensitivity, providing children with a sense of emotional security and protection. Various researchers highlight the fundamental importance of the attachment relationship between parents and children for the child’s adaptation, beginning from the early years of life [5]. Subsequent studies have reaffirmed the significance of secure attachment in emotional regulation and the construction of a positive self-perception in adulthood [6,7]. The findings of Clark and Symons [8] indicate that establishing secure attachment is essential for children to experience a realistic and comfortable self-perception. Likewise, it has been evidenced that secure attachment patterns have a positive impact on social relationships and more adaptive aspects of self-image [9]. Self-concept refers to an individual’s perception and understanding of themselves, encompassing their physical characteristics, abilities, social roles, beliefs, and values. It is a cognitive construction that influences how we perceive, evaluate, and behave in various life situations. The self-concept can comprise diverse dimensions, such as self-image (how we perceive ourselves physically), self-esteem (how we assess our own value), the skills and competencies we perceive ourselves to possess, and beliefs about our personal qualities. In this vein, the significance of positive parenting and secure attachment relationships has been emphasized in laying the foundation for healthy adaptation and optimal psychological development throughout life [10], and secure attachment has a positive influence on life satisfaction [11].

Secure attachment provides a solid foundation for resilience in the face of adversity. Grotberg [12] states that resilience is an inherent capacity in all human beings to confront the difficulties and challenges of life, to successfully cope with them, or even to be positively transformed by them. It is not an isolated individual characteristic but a combination of internal and external factors. Three factors are associated with resilience: individual characteristics such as self-efficacy and self-esteem, family support, and support from someone outside the family [13,14]. Thus, children with secure attachment tend to develop greater ability to cope with stressful situations and recover from traumatic experiences; it acts as an emotional buffer that enables them to face life’s challenges with greater effectiveness and adaptability [13,15,16]. Furthermore, the most resilient children and adolescents come from families with better functioning and receive more guidance and supervision from their parents and other family adults [17].

Secure attachment is closely related to positive self-esteem, particularly with the presence of secure support in close relationships [4]. Positive self-esteem refers to a person’s positive perception and evaluation of themselves [18]. It involves having a self-image as someone deserving of love, respect, and consideration, and feeling competent and capable in various areas of life. In this context, research has explored the “secure-base script”, an internal mental model related to secure attachment, and how it can influence a person’s positive self-esteem [6], or how peer relationships and perceptions of security in the mother–child relationship are related to self-esteem [19]. Furthermore, the stability of self-esteem over time has been demonstrated, suggesting that early experiences of secure attachment can have a lasting impact on a person’s self-esteem [20].

Despite the relationship between attachment and self-concept, as well as with resilience and positive self-esteem, having been the subject of research with significant findings, a lack of clear evidence regarding the specific role of resilience and positive self-esteem in the connection between attachment security and self-concept has been identified. This research aims to fill this gap, highlighting the importance of understanding how parenting processes, particularly an appropriate model of attachment security, influence the development of self-concept in individuals. However, for a deeper understanding of this relationship, it is essential to consider the mediating role of resilience and positive self-esteem. It is postulated that well-developed resilience and positive self-esteem can act as protective factors, enabling individuals to effectively and adaptively face life’s challenges and, in turn, influence the formation and consolidation of a more solid and positive self-concept. Thus, the complex interaction between attachment security, resilience, and positive self-esteem may be crucial for a more comprehensive understanding of identity development and self-perception in the context of parenting experiences.

In essence, parenting significantly impacts personal development, such that secure attachment content influences coping traits and adult perceptions. Secure attachment plays a role in shaping how we approach challenges, crises, and traumas in the future. Rooted in secure upbringing, the capacity to effectively confront reality is heightened, as coping strategies have been comprehended and practiced as a developmental process. Furthermore, this mode of engaging with reality impacts emotional sentiments and evaluative perceptions, shaping one’s sense of self-worth. Ultimately, these relationships may contribute to a deeper understanding of how attachment models influence self-assessment of one’s traits, abilities, beliefs, and values, stemming from a model guided by the following research inquiries: What is the implication of perceived attachment security and self-concept in adults? Do resilience and positive self-esteem possess separate explanatory capacities to enhance the comprehension of this relationship? Is there a sequential mediating role of resilience and positive self-esteem that aids in comprehending the interplay between attachment and self-concept?

## 2. Literature Review

### 2.1. Secure Attachment and Self-Concept

The relationship between secure attachment and self-concept is a complex interaction that has been addressed by various authors in the scientific literature. Researchers have investigated how secure and insecure attachment styles in childhood are related to the formation of self-concept in adolescence and have found that adolescents with secure attachment tend to develop a more positive and coherent self-concept [21]. In this regard, Maunder and Hunter [22] have examined how early experiences of secure attachment influence emotional self-regulation and the perception of oneself as competent and capable in social and emotional situations. Emotional self-regulation is understood as an individual’s ability to recognize, comprehend, and manage their own emotions effectively.

Paying attention to self-concept becomes an essential task, especially during adolescence, where it intertwines with the assertion of identity, decision making, and the search for independence and autonomy [23]. Parental and family bonds exert a significant influence on the development of self-concept [24], and this is manifested through various modes of parental socialization [25], the type of communication between parents and children [26], the style of affectionate upbringing [27], or the family atmosphere [28].

Higgins’ theory of self-concept regulation has also highlighted how experiences of secure attachment influence the way individuals process and organize information about themselves, affecting the coherence and stability of self-concept [29]. In this line of research, studies have shown how attachment styles affect self-esteem and self-perception in situations of intimacy and emotional closeness [30].

These studies suggest that secure attachment in infancy plays a fundamental role in the formation and development of self-concept throughout life. The quality of early relationships with primary caregivers influences how individuals perceive themselves, develop a positive self-image, and maintain coherence and stability in their personal identity. From this perspective, the present study develops the following hypothesis:

**H1.**  *Perceived security is positively related to self-concept*.

### 2.2. Secure Attachment, Resilience, and Self-Concept

Secure attachment has a significant impact on the development of resilience in individuals, which in turn influences self-concept. It has been demonstrated that the quality of secure attachment in infancy is positively associated with higher levels of resilience in adulthood [31]. Secure attachment provides a foundation for individuals to cope with adverse situations, enabling them to develop greater self-confidence and belief in their abilities to confront and overcome challenges (resilience). Additionally, Masten and Reed suggested that resilience is a dynamic process that can be modified and strengthened throughout life, even in response to negative experiences [32]. This implies that secure attachment in infancy not only promotes the initial development of resilience but can also continue to nourish and enhance this adaptive capacity in later stages of life.

Likewise, resilience has a direct impact on individuals’ self-concept. Studies have revealed that individuals with higher levels of resilience tend to have a more positive self-image and greater self-confidence [33]. The ability to effectively cope with challenges and overcome adversities reinforces a positive perception of oneself as capable and competent individuals. In this regard, research by Infurna and Luthar has shown that resilience protects against the development of negative self-esteem, even during times of stress or difficulties [34]. Thus, resilience acts as a protective factor that mitigates the negative effects of adverse experiences on self-concept, enabling individuals to maintain a positive and healthy self-image.

Secure attachment plays an essential role in the development of resilience, which in turn influences individuals’ self-concept. Early experiences of secure attachment provide a solid emotional foundation for the development of resilience throughout life, which, in turn, contributes to a more positive self-image, greater self-confidence, and, consequently, fewer maladaptive schemas [35]. Considering the aforementioned findings, the following hypothesis was proposed in the study:

**H2.**  *The relationship between security and self-concept is mediated by resilience*.

### 2.3. Secure Attachment, Positive Self-Esteem, and Self-Concept

Secure attachment and self-esteem have been extensively investigated from various theoretical perspectives and by different authors. Self-determination theory has highlighted the importance of secure attachment in satisfying basic psychological needs, such as autonomy, competence, and relatedness to others [36]. Self-determination refers to an individual’s ability to act in accordance with their own choices, values, and internal goals, rather than being directed by external influences or social pressures. Secure attachment experiences can influence how individuals compare themselves to others and how they perceive themselves in terms of competence and personal worth, thereby affecting their self-esteem and self-concept [37]. On one hand, secure attachment provides a foundation for individuals to develop a sense of autonomy and competence in their relationships with others, which can have a positive impact on self-esteem [38], as children learn to perceive themselves as competent and capable [39]. On the other hand, secure attachment provides a foundation for individuals to develop adaptive coping and emotional regulation strategies, contributing to a more positive self-esteem and a more coherent and stable self-perception [40].

Erikson’s theory of identity highlights that the formation of self-concept and personal identity are critical tasks in adolescence. Secure attachment facilitates the development of a positive and coherent identity in adolescents, influencing their self-esteem and self-perception [41]. In line with this, previous studies have shown that individuals with secure attachment tend to have higher levels of self-esteem. Wu examined the mediating effect of self-esteem on the relationship between attachment tendencies and self-concept clarity, using a path model, and found that individuals with secure attachment have higher self-esteem, leading to greater self-concept clarity [42]. Similarly, the positive relationship between security scores and self-concept has been demonstrated [43], as well as the connection between attachment-based parenting models and identity-related psychological characteristics and social relationships of individuals, indicating that attachment security is associated with self-esteem and self-concept as individuals grow [8].

Similarly, a more positive self-concept can influence the strengthening of secure attachment, as formulated by Steele in his theory of self-affirmation, where he suggests that individuals have an intrinsic motivation to maintain a positive self-image and preserve coherence between their beliefs and behaviors [44]. A more positive self-concept can influence the perception of relationships with others and affect expectations of support and affection in social interactions, which can lead to a higher likelihood of establishing secure attachment relationships [38].

Secure attachment and self-esteem are interconnected through multiple theories and psychological processes. Experiences of secure attachment in childhood and adolescence provide a solid emotional foundation for individuals to develop positive self-esteem, which in turn influences the formation and consolidation of self-concept throughout life. Considering the previous findings, a third hypothesis was formulated for this research:

**H3.**  *The relationship between security and self-concept is mediated by positive self-esteem*.

### 2.4. Secure Attachment, Resilience, Positive Self-Esteem, and Self-Concept

Secure attachment provides a solid emotional foundation for the development of resilience and positive self-esteem [3], which, in turn, can influence individuals’ self-perception and the construction of their self-concept throughout life. It is known that higher resilience can strengthen self-esteem and, in turn, enhance self-concept [45]. Furthermore, resilience, as the capacity to face and overcome adversities, may promote greater confidence in one’s abilities and resources, which is associated with a more positive self-esteem [13]. Conversely, positive self-esteem can influence individuals’ self-perception and how they interpret social experiences, thereby impacting the formation and consolidation of self-concept [46].

Similarly, studies have examined how self-esteem can mediate the relationship between secure attachment and self-concept in young adults [47]. Secure attachment in childhood may promote higher self-esteem in adulthood, which in turn influences how individuals see themselves and how they perceive their relationships with others and the world around them. Based on the previous findings, a fourth hypothesis was considered for this research:

**H4.**  *The relationship between security and self-concept is sequentially mediated by resilience and positive self-esteem*.

## 3. Materials and Methods

### 3.1. Research Design

A quantitative, cross-sectional, and nonprobabilistic research design was employed to examine the role of resilience and positive self-esteem in the relationship between childhood security and current self-concept.

### 3.2. Participants

Initially, 407 responses were obtained randomly. After the data cleansing process, the final study participants (*n* = 383) consisted of adults aged between 17 and 86 years (M = 28.52 years, SD = 12.23 years), with 76.2% being females and 66.1% having completed university studies. Additionally, 35.8% of the participants reported living with both parents, with or without siblings (see Table 1).

### 3.3. Measures

For perceived security, the reduced version of the CaMir questionnaire (CaMir-R) was used to measure attachment representations. Specifically, the shortened version consists of 32 items, rated on a 5-point Likert scale (ranging from 1 = completely disagree to 5 = completely agree), and assesses security, family concern, parental interference, self-sufficiency, and childhood trauma [48]. For the present study, the factor labeled security (related to the availability and support of attachment figures) was used, comprising 7 items (e.g., “When I was a child, my loved ones made me feel that they enjoyed spending time with me”), which refers to the perception of having felt loved by attachment figures, being able to trust them, and knowing their availability. It is associated with secure attachment and demonstrates high internal consistency (α = 0.85).

For self-concept, the AF-5 Self-Concept Scale [49] was employed. This 30-item scale (e.g., “I get scared easily”) assesses 5 dimensions of self-concept (6 items per dimension): social, academic/professional, emotional, family, and physical. Respondents use a 5-point Likert scale (ranging from 1 = completely disagree to 5 = completely agree). Specifically, the overall or total dimension of self-concept was used, demonstrating high internal consistency (α = 0.84).

For resilience, Connor and Davidson’s Resilience Scale (CD-RISC) [50] was used, specifically the reduced version (CD-RISC10) developed by Campbell-Sills and Stein [51] and adapted to Spanish by Notario-Pacheco et al. [52]. The original instrument consists of 25 items responded on a 5-point Likert scale (0 = not at all to 4 = almost always), comprising five factors: persistence–tenacity–self-efficacy; tolerance of negative affect; adaptability and coping; positive acceptance of change; and spiritual influences. The reduced version (CD-RISC10) measures global resilience and consists of 10 items (e.g., “I am able to adapt to changes”). Higher scores indicate greater capacity to overcome traumatic circumstances.

For self-esteem, the Rosenberg Self-Esteem Scale [18], validated in Spanish by Martín-Albo et al. [53], was administered. It consists of 10 items (e.g., “Overall, I am satisfied with myself”) rated on a 5-point Likert scale (ranging from 1 = strongly disagree to 2 = strongly agree). The scale assesses feelings of self-acceptance and self-respect, capturing how individuals value themselves. Scores are differentiated into positive and negative self-esteem. In the current study, positive self-esteem was utilized, and its score was computed as the mean of the five items related to positive self-esteem.

Finally, the sociodemographic block of questions was included. The variables used were age (continuous variable, in years completed); gender (dichotomous variable, male or female); educational level (polytomous variable with five response categories: primary studies or less; secondary studies or basic training; vocational training; high school studies; and university studies); and, finally, the type of family life (polytomous variable with 6 categories: live alone; live with friends; single parent family; heteroparental family without children; heteroparent with children; others).

### 3.4. Procedure

The questionnaire with the different scales was created online. Prior to its dissemination, the form was pilot-tested with a group of researchers to detect any operational errors or issues in question interpretation. Following this verification, the field work was carried out by three university students trained to implement the study through physical and digital social networks.

The questionnaire’s header contained the research objective, and anonymity was ensured. Prior informed consent was obtained before its implementation, following the ethical approval of the study. The responses were consolidated into a common database for data cleansing and analysis. Fieldwork was conducted in February and March of the year 2023, with an average completion time of 17 min per questionnaire. The questionnaire was self-administered, freely, without compensation such as prizes, rewards, or contests.

### 3.5. Data Analysis

Firstly, the scores for the scales were computed by taking the mean of the different validated factors. The security factor of the CaMir-R scale was calculated to assess perceived security, using the weighted average of the seven variables that comprise it. The total autoconcept was derived by calculating the mean of each of the specific autoconcept dimensions (family, emotional, occupational, social, and physical). For positive self-esteem, the mean of the first 5 items from the Rosenberg self-esteem scale was used. Lastly, the mean of the 10 items from the resilience scale was calculated.

Secondly, the database was cleaned and checked for accuracy, ensuring the absence of missing values. Concerning multivariate outliers, cases were identified and eliminated using three criteria: the Mahalanobis distance index, the Cook’s distance index, and leverage [54]. Two out of the three criteria were employed to identify outliers (*n* = 24). According to the Mahalanobis distance, values were considered outliers if they exceeded the critical chi-square value for three predictor variables (df 3), with *p* = 0.001, x^2^ = 16.27 (*n* = 9). According to the Cook’s distance criterion, outliers were implied when the values were greater than Di = 0.0099 (*n* = 28). Lastly, according to the leverage criterion, values were considered outliers if they exceeded twice the number of predictors plus two, scaled by the sample size, h_ii_ = 0.0197 (*n* = 34).

Thirdly, a descriptive analysis of the study variables was conducted, reporting the mean, standard deviation, percentage, skewness, and kurtosis. Additionally, a correlational analysis was performed to identify significant associations between the variables included in the mediation model (perceived security, self-concept, resilience, and positive self-esteem), assessing the effect size of the correlation coefficient based on Cohen’s criteria [55]: small (0.10), medium (0.30), or large (0.50).

Finally, to examine the role of resilience and positive self-esteem in the relationship between perceived security and self-concept, a mediation analysis was conducted, a relevant approach in psychosocial studies [56]. The sequential mediation model was tested to estimate the effects, using the method with 10,000 bootstrap samples to correct for bias and estimate the 95% confidence interval (CI), ensuring statistically robust and precise results [57]. The calculations were performed using the PROCESS macro (Model 6) in the Statistical Package for the Social Sciences (SPSS) [58], with perceived security as the predictor variable, self-concept as the outcome variable, and resilience and positive self-esteem as the mediating variables. All analyses were considered significant at a *p*-value less than 0.05.

## 4. Results

### 4.1. Descriptive and Correlational Analysis

Table 2 shows the descriptive statistics and the correlations. The skewness values (between −1.47 of perceived security and −0.33 of self-concept) and kurtosis values (between −0.44 of resilience and 1.72 of the perceived security) of the study variables were considered appropriate. Mean levels of perceived security (M = 4.23, SD = 0.91) and self-concept (M = 3.64, SD = 0.52) were reported. Correlation analysis revealed significant positive associations between all study variables. The strongest and large positive relationship was found between positive self-esteem and self-concept (r = 0.67, *p* < 0.001). It was also reported that the relationship between the independent variable (perceived security) and the outcome variable (self-concept) is positive and large (r = 0.58, *p* < 0.001).

### 4.2. Sequential Mediation Model

**Figure 1:**
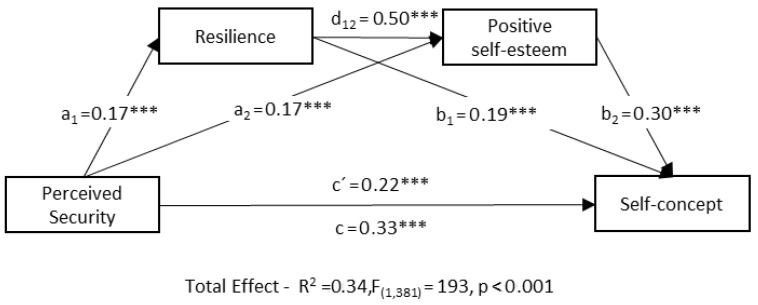
Sequential mediation model of resilience and positive self-esteem in the relationship between perceived security and self-concept. Notes: Indirect effect through resilience (a_1_*b_1_, b = 0.03, SE = 0.01, CI 95% [0.015, 0.054]; indirect effect through positive self-esteem (a_2_*b_2_, b = 0.05, SE = 0.01, CI 95% [0.030, 0.079]; indirect effect through perceived security and resilience (a_1_*d_12_*b_2_, b = 0.02, SE = 0.01 CI 95% [0.013, 0.039]. *** *p* < 0.001.

The results of the sequential mediation model, examining the effect of resilience and positive self-esteem on the relationship between perceived security and self-concept, are presented in Figure 1 and Table 3.

All simple mediation relationships were statistically significant (Figure 1 and Table 3). Perceived security (predictor variable) had a significant and positive effect on resilience (b = 0.17, t = 4.44, *p* < 0.001, 95% CI [0.093, 0.240]) and positive self-esteem (b = 0.17, t = 5.66, *p* < 0.001, 95% CI [0.112, 0.231]). For each unit increase in perceived security, there was an increase in the level of resilience and positive self-esteem. Moreover, self-concept (outcome variable) was positively and significantly predicted by positive self-esteem (b = 0.30, t = 9.76, *p* < 0.001, 95% CI [0.242, 0.364]) and resilience (b = 0.19, t = 6.72, *p* < 0.001, 95% CI [0.137, 0.251]). For each unit increase in positive self-esteem or resilience, there was an increase in self-concept. The direct effect between the two mediating variables (resilience on positive self-esteem) showed a significant and positive relationship (b = 0.50, t = 12.36, *p* < 0.001, 95% CI [0.419, 0.578]). Thus, for each unit increase in the level of resilience, there was an increase in positive self-esteem.

As a result, all indirect effects showed a significantly direct relationship (Table 3). Individually, there is indirect mediation through each mediating variable. Thus, there is an indirect effect between perceived security and self-concept both through resilience (b = 0.03, SE = 0.01, 95% CI [0.01, 0.05]) and positive self-esteem (b = 0.05, SE = 0.01, 95% CI [0.030, 0.079]). Moreover, a sequential indirect effect was found through both mediators (b = 0.02, SE = 0.01, 95% CI [0.013, 0.055]). Additionally, a significant direct relationship was found with the total of indirect effects (b = 0.11, SE = 0.02, 95% CI [0.074, 0.148]). The mediation model accounts for 34% of the variability in self-concept.

The comparison of the strength of the indirect effects through pairwise contrasts (see Table 3) reveals that the simple indirect effect of positive self-esteem is greater than the sequential indirect effect (b = 0.03, SE = 0.01, 95% CI [0.005, 0.095]). However, no significant differences were found in the rest of the comparisons of indirect effects.

Finally, the results suggest the presence of a partial and complementary mediating effect. The path analysis revealed that perceived security during childhood is related to adult self-concept. However, this perception of security also exerts effects on both resilience and positive self-esteem, which collectively contribute to a more comprehensive understanding of its effect on self-concept.

## 5. Discussion

The current study aimed to explore the association between parenting aspects and their influence on adult individuals, within the context of family studies, which has garnered considerable attention in the research field. More specifically, this cross-sectional investigation focused on examining the influence of perceived security (as an indicator of secure attachment) on both resilience and positive self-esteem, as these factors play a crucial role in shaping an individual’s self-concept.

The findings suggest a positive association between perceived security and self-concept, supporting hypothesis H1. Similar results have been highlighted in other studies, such as those reported by Kaur et al. [59], indicating a positive correlation between a supportive family environment, security, and a positive self-concept. Additionally, Kashif et al. [60] found a significant relationship between good home quality, secure attachment, and a positive self-concept, while Maunder and Hunter [22] reported similar findings. According to Torres and Rodrigo [61], there exists a link between attachment and self-concept, both being important and fundamental dimensions in predicting the psychological wellbeing of children and adolescents, thus reinforcing the existing evidence between these two constructs.

In line with this, the relationship between perceived security and self-concept is also mediated by resilience, confirming hypothesis H2. Thus, this mediating variable provides a better understanding of the link between attachment and self-concept, as observed in studies by Chentsova et al. [62], examining attachment, resilience, and self-concept in adolescents; García-Martínez et al. [63], highlighting the significance of self-concept in relation to resilience and parental support; and other studies conducted by Pilowsky et al. [64] and Sakyi et al. [65], among others. These studies reinforce the idea that resilience plays a mediating role in the relationship between perceived security in close relationships and the development of positive self-concept, providing deeper insights into how these factors interact to influence one’s perception of themselves.

According to Bowlby’s [3] and Ainsworth’s [4] attachment theory, humans have an innate need to form emotional bonds with attachment figures, especially during early years of life. The quality of these early relationships affects how individuals perceive and cope with the world throughout their lives. Perceived security in attachment provides a secure base from which an individual can explore the world and develop a more positive self-image. In turn, higher resilience may arise from secure attachment, as supportive and caring experiences in childhood lay a solid foundation for coping with challenges and adversities in life. Consequently, the self-concept will be optimally influenced by adequate levels of resilience, where positive attachment is indicative of personal capacity to confront adversity. Thus, the type of attachment influences resilience and, therefore, self-concept.

Regarding the role of self-esteem, it also acts as a mediator in the relationship between parenting style and self-concept, supporting hypothesis H3. Research by Belsky and Jaffee [66], McLeod et al. [67], and Barber [68] has established this association. This implies that self-esteem also significantly influences self-concept [43,69], and it is influenced by the perception of attachment security. Thus, this perception of security influences self-esteem, and self-esteem, in turn, affects self-concept. These studies provide evidence of how perceptions of parenting and parent–child relationships can influence individuals’ self-esteem and, consequently, impact the formation and development of self-concept. Self-esteem plays a significant mediating role in this relationship, as the ways in which parents interact with their children can shape their self-perception and self-concept over time.

From a general perspective, hypothesis H4 is also confirmed, as the results provide evidence of how resilience and self-esteem mediate sequentially in the relationship between perceived security and self-concept. This finding contributes to the knowledge in family studies and, specifically, to the understanding of the link between parenting development and individuals’ psychosocial evolution. These results align with the notion that secure attachment provides a secure foundation from which individuals can develop self-confidence and the ability to cope with life challenges [70]. Furthermore, self-determination theory posits that self-esteem and resilience are key factors in developing a positive identity and a healthy self-perception [36]. Previous studies have highlighted the importance of the individual mediator variables separately, but the present study contributes to clarifying their combined and sequential effects.

It is also important to highlight the role of the simple mediation of self-esteem, which emerges as the most prominent relationship in the model. In other words, to better understand how a parenting model affects the cognitive aspect of self-perception, it is necessary to include the emotional component of how we feel about ourselves, as evidenced in the study by Cui et al. [71]. This does not diminish the role of resilience; on the contrary, it allows us to see how parenting (perceived security) influences the way we think about ourselves (self-concept), but the significant influence of the emotional component (self-esteem) is related to how we can also cope with critical situations in life (resilience).

The findings of this study can be interpreted considering the positive parenting approach, supported by previous research. For instance, Kochanska and Kim [72] found that positive parenting is associated with a higher sense of secure attachment in children, highlighting the importance of affectionate and sensitive parenting for the development of perceived security in childhood. Additionally, studies such as Collins and Feeney [73] indicate that positive parenting is related to a more positive self-concept in children, while Cornellà-Font et al. [43] demonstrated its link to higher self-esteem and a more favorable self-concept in adolescents. In line with this, the family systems theory emphasizes the influence of family functioning on the development of individuals’ resilience [74], and the Recommendation of the Council of Europe on Positive Parenting [1] supports that positive parenting approach promotes resilience in children, strengthening their ability to cope with stressful situations. Consequently, these results provide a deeper understanding of the mechanisms through which self-concept can be influenced and suggest the promotion of strategies that foster mental wellbeing, improved academic performance, healthy family relationships, and more positive social coexistence.

It is also necessary to specify the high correlation between self-esteem and self-concept. One might even interpret that the same parameters are being assessed. However, various authors support a multidimensional and hierarchical view of self-concept, encompassing both cognitive aspects pertaining to self-image and affective aspects linked to self-esteem. Research such as that by Marsh and Craven [75] suggests that self-esteem is more transient, contingent on context, and more unstable than the more specific components of self-concept (educational, social, familial, emotional, and physical). This underscores a substantial correlation between both concepts. Nonetheless, their inclusion in the study is imperative to ascertain whether the more emotional or affective perception plays a significant role in the more cognitive perception of the individual, within a broader framework of knowledge, as proposed by the sequential mediation model.

However, there are some limitations in the present study. Firstly, more heterogeneous samples would be needed to relate structural and cultural variables. This could contribute to the theoretical development presented by seeking models based on invariance. Secondly, despite the theoretical justification of the model, the use of a cross-sectional research design limits the interpretation of causality. Longitudinal studies are required to enhance the predictive capacity of the model. Lastly, factors that may influence individual development and could affect the model were not included. For instance, the experience of traumatic events in individuals may moderate the proposed relationships between parenting and psychological characteristics as adults.

Despite these limitations, this research significantly contributes to the literature, particularly in family studies that relate parenting conditions to psychosocial aspects in adults. It also paves the way for future research, such as the utilization of other psychometric resources related to personality traits in the design of models, or the inclusion of the influence of family and social context as moderator variables of the existing relationships from a systemic perspective.

## 6. Conclusions

The present study highlights that, compared to the direct effect of perceived security on self-concept, a greater predictive capacity is observed when considering the sequential mediation of resilience and positive self-esteem. Thus, the findings suggest that the ability to cope with adversity and personal acceptance underlie the impact of perceived security on self-concept. Moreover, the benefits of the study demonstrate that the separate mediating effects of both resilience and positive self-esteem enhance the causality between perceived security and self-concept. In other words, parenting influences the cognitive manner in which we perceive ourselves as adults. However, this relationship is better understood when considering the impact that parenting also has on our ability to cope with adversity, as well as the emotional and subjective perception we hold of ourselves.

These results not only contribute to a deeper understanding of family studies that link the parenting process to developmental psychological characteristics but also have implications for public policies. The development of family interventions should consider promoting positive parenting and, consequently, the development of secure attachments. These intervention models align with the approach of positive parenting. Additionally, in socioeconomically and family disadvantaged conditions, intervention programs for adolescents and young individuals should include content aimed at fostering resilience and self-esteem. This will help improve self-concept in their personal development. Therefore, these findings have significant implications for intervention and support for parents, educators, and professionals, as they emphasize the importance of secure and affectionate parenting for the healthy psychosocial development of individuals. Promoting resilience and self-esteem can be an effective approach to strengthen identity and emotional wellbeing, enabling individuals to positively cope with life’s challenges and fostering a more positive perception of themselves.

In conclusion, the exercise of parenting is of vital importance for the developmental growth of individuals. The quality of parenting influences the capacity to face future challenges and also plays a crucial role in emotional and affective evaluation, facilitating an optimal self-assessment characterized by acceptance, respect, and appreciation. This, in turn, results in high levels of effective cognitive self-description.

## Figures and Tables

**Table 1:** Sociodemographic characteristics of the study sample.

Variables	Categories	*n* = 383
Age (Years)	Mean (SD)	28.52 (12.23)
Sex	Men	91 (23.8%)
*n* (%)	Women	292 (76.2%)
Education level	Primary studies or less	8 (2.1%)
*n* (%)	Secondary studies or basic training	20 (5.2%)
	Vocational training	65 (17%)
	High school studies	37 (9.6%)
	University studies	253 (66.1%)
Type of family life	Live alone	19 (5%)
*n* (%)	Live with friends	25 (6.5%)
	Single parent family	108 (28.2%)
	Heteroparental family without children	40 (10.4%)
	Heteroparent with children	177 (46.2%)
	Others	14 (3.7%)

**Table 2:** Descriptive statistics and correlation between measured variables.

Variables	M	SD	A	K	1	2	3	4
1 Perceived security	4.23	0.91	−1.47	1.72	-			
2 Self-concept	3.64	0.52	−0.33	−0.01	0.58 ***	-		
3 Resilience	3.93	0.68	−0.45	−0.44	0.22 ***	0.56 ***	-	
4 Positive self-esteem	4.28	0.66	−0.81	0.29	0.35 ***	0.67 ***	0.57 ***	-

Note. M = mean; SD = standard deviation; A = asymmetry; K = Kurtosis. *** *p* < 0.001.

**Table 3:** Model summary information and the direct, indirect, and total effects for serial mediation of resilience and positive self-esteem between perceived security and self-concept.

	Resilience (M1)			Positive Self-Esteem (M2)			Self-Concept (Criterion Variable)		
**Direct Effects ^1^**	**b**	**SE**	**t**	**b**	**SE**	**t**	**b**	**SE**	**t**
Constant	3.23	0.16	19.68	1.60	0.18	8.78	0.65	0.12	5.40
PS	0.17	0.04	4.44	0.17	0.03	5.66	0.22	0.02	11.57
RE	-	-	-	0.50	0.04	12.36	0.19	0.03	6.72
PSE	-	-	-	-	-	-	0.30	0.03	9.76
	F_(1,381)_ = 19.68, *p* < 0.001, R^2^ = 0.05			F_(2,380)_ = 113.55, *p* < 0.001, R^2^ = 0.37			F_(3,379)_ = 212.35, *p* < 0.001, R^2^ = 0.63		
**Total effects ^1^**									
Constant	-	-	-	-	-	-	2.25	0.10	21.96
PS	-	-	-	-	-	-	0.33	0.024	13.89
							F_(1,381)_ = 193, *p* < 0.001, R^2^ = 0.34		
**Indirect Effects**		**Effect**		**BootSE**		**Boot-LLCI**		**Boot-BULCI**	
Total		0.11		0.02		0.07		0.15	
Ind1 PS→RE→SC		0.03		0.01		0.01		0.05	
Ind2 PS→PSE→SC		0.05		0.01		0.03		0.08	
Ind3 PS→RE→PSE→SC		0.02		0.01		0.01		0.04	
Contrast indirect effects									
C1 (Ind1-Ind2)		−0.02		0.02		−0.05		0.01	
C2 (Ind1-Ind3)		0.01		0.01		−0.01		0.02	
C3 (Ind2–Ind3)		0.03		0.01		0.01		0.05	

Note. ^1^ *p*-value of all effects is *p* < 0.001. PS = perceived security, RE = resilience, PSE = positive self-esteem, SC = self-concept. M1 = first mediator, M2 = second mediator.

## Data Availability

The raw data supporting the conclusions of this article will be made available by the authors, without undue reservation, upon request.
